# Drug Abuse Problems are Associated with Poorer Health-Related Quality of Life

**DOI:** 10.1192/j.eurpsy.2026.10494

**Published:** 2026-07-31

**Authors:** Y. Jung, M.-D. Kim

**Affiliations:** Jeju National University, Jeju, Korea, Republic Of

## Abstract

**Introduction:**

Drug abuse is defined as the harmful or hazardous use of psychoactive substances, including illicit drugs. Drug abuse is associated with a range of negative outcomes affecting physical health, psychological well-being, social functioning, and environmental stability, all of which collectively impact an individual’s health-related quality of life (HRQOL). Previous studies have reported that individuals undergoing treatment for drug addiction exhibit significantly lower HRQOL compared to the general population. Among those receiving treatment, improvements in quality of life have been linked to reduced relapse rates.

**Objectives:**

Evaluation of HRQOL in the context of drug abuse should consider cultural variability, and research specific to Asian populations remains limited. Therefore, this study investigated the relationship between HRQOL and drug abuse-related problems in a community sample in Korea.

**Methods:**

Data were collected from 500 community-dwelling adults residing in Jeju, Korea. Participants were asked whether they had used opiate (narcotic) analgesics, diet drugs, or illicit drugs during the past 12 months, with responses recorded as “yes” or “no.” Participants who responded “yes” to any of these categories subsequently completed the Drug Abuse Screening Test–10 (DAST–10). HRQOL was measured using the 26-item abbreviated version of the World Health Organization Quality of Life instrument.

**Results:**

In total, 25 participants (5.0%) reported experiencing drug abuse problems within the previous 12 months. Univariate analyses revealed that lower scores across all WHOQOL–BREF domains were significantly associated with the presence of drug abuse problems. Additionally, older age, male sex, lower educational attainment, and lower monthly household income were significantly associated with lower scores in the WHOQOL–BREF domains. Higher depressive symptom severity, as measured by PHQ-9 scores, was also significantly correlated with lower scores in all WHOQOL–BREF domains (Table 1). The results of the multivariate regression analyses are presented in Table 2. After adjusting for relevant covariates, including age group, sex, education, monthly household income, and depressive symptoms, drug abuse problems remained independently associated with lower scores in all WHOQOL–BREF domains.

**Image 1:**
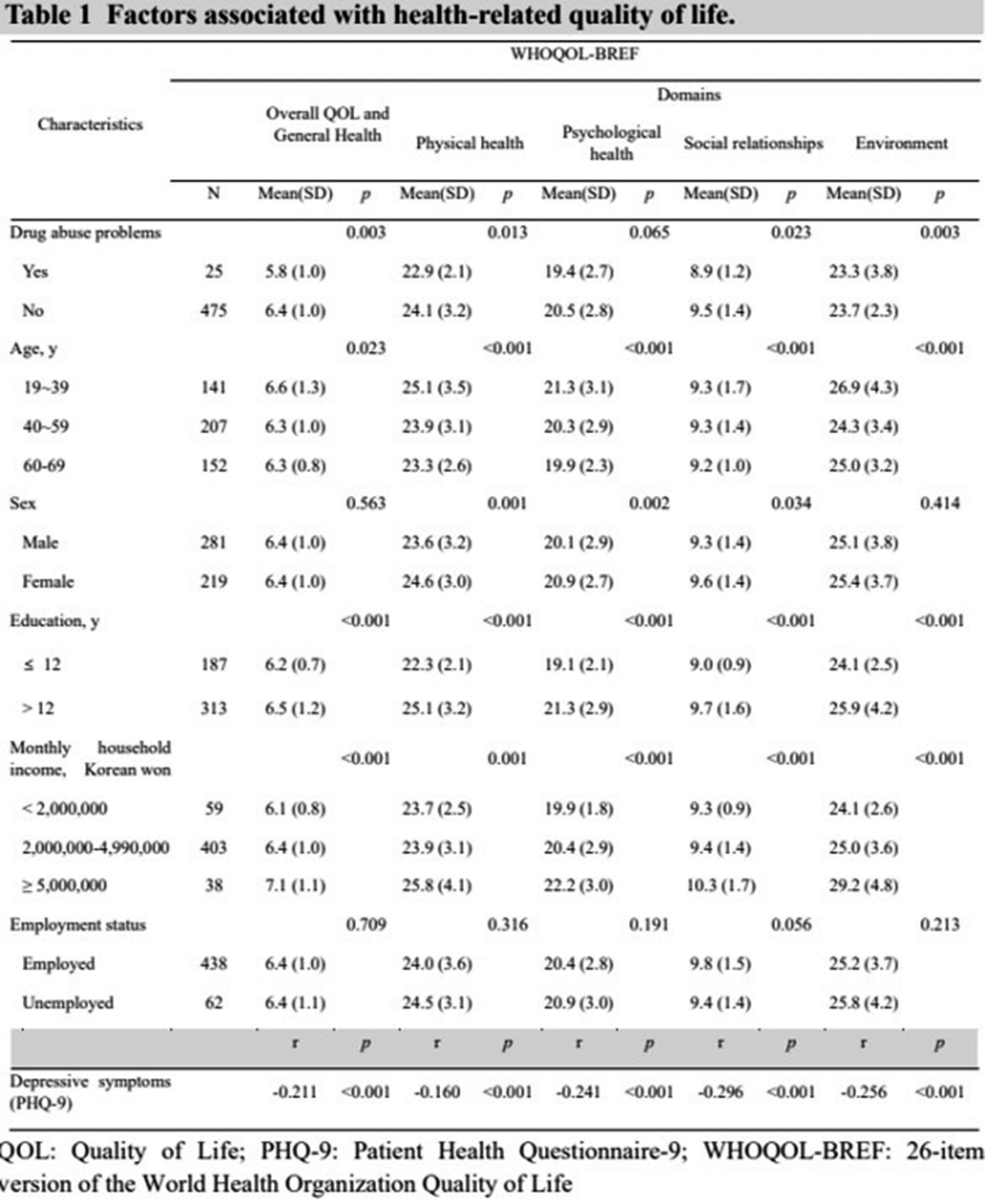
Image 1: Long description.

**Image 2:**
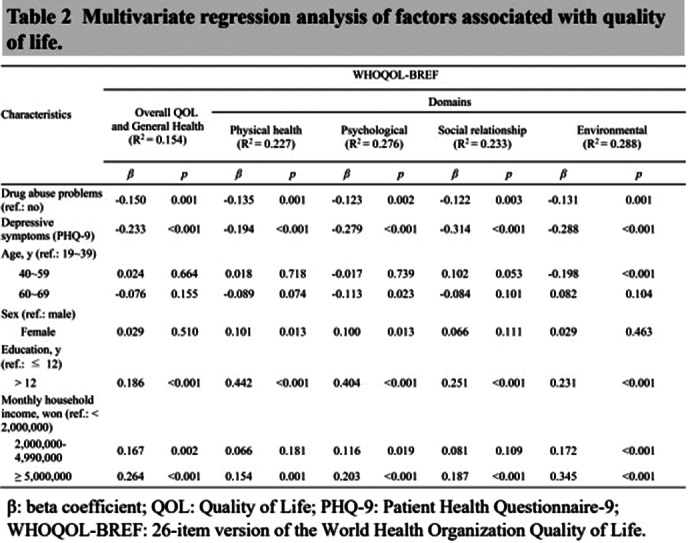
Image 2: Long description.

**Conclusions:**

This study demonstrated a significant association between drug abuse problems and reduced HRQOL among adults in a Korean community setting. These findings highlight the importance of integrated prevention and treatment strategies that address not only the physical and psychological aspects of drug abuse but also the broader social context. Despite its limitations, this study provides valuable baseline data that can inform the development of community-specific interventions aimed at improving overall well-being in populations at risk for substance abuse.

**Disclosure of Interest:**

None Declared

## Long descriptions

### Image 1: Long description

The table header identifies the instrument as WHOQOL-BREF. The columns are divided into Characteristics, N, and five outcome domains: Overall Q O L and General Health, Physical health, Psychological health, Social relationships, and Environment. Each domain reports Mean (S D) and p-values.

Row groups include:

* Drug abuse problems: Yes (N=25) and No (N=475). Significant p-values for Overall Q O L (0.003), Physical (0.013), Social (0.023), and Environment (0.003).

* Age, y: Groups 19-39, 40-59, and 60-69. All domains except Overall Q O L show highly significant differences (p < 0.001).

* Sex: Male (N=281) and Female (N=219). Significant differences in Physical (0.001), Psychological (0.002), and Social (0.034) domains.

* Education, y: Less than or equal to 12 and greater than 12. All domains show highly significant differences (p < 0.001).

* Monthly household income, Korean won: Three tiers from < 2,000,000 to >= 5,000,000. All domains show significant differences (p <= 0.001).

* Employment status: Employed and Unemployed. No significant differences across any domains (p > 0.05).

* Depressive symptoms (P H Q-9): A bottom row showing Pearson correlation coefficients (r) and p-values. All correlations are negative, ranging from -0.160 to -0.296, with all p-values < 0.001.

Footnotes define Q O L as Quality of Life, P H Q-9 as Patient Health Questionnaire-9, and WHOQOL-BREF as the 26-item version of the World Health Organization Quality of Life scale.

Navigate back to Image 1:.

### Image 2: Long description

The table presents data for W H O Q O L B R E F across five main columns. Overall Q O L and General Health (R super 2 = 0.154), Physical health (R super 2 = 0.227), Psychological (R super 2 = 0.276), Social relationship (R super 2 = 0.233), and Environmental (R super 2 = 0.288). Each domain is split into beta coefficient and p-value columns.

Key findings by characteristic row:

* Drug abuse problems (ref. no): Shows negative beta coefficients across all domains, ranging from -0.122 to -0.150, all with p < 0.005.

* Depressive symptoms (P H Q 9): Shows the strongest negative associations across all domains, with beta values between -0.194 and -0.314, all with p < 0.001.

* Age, y (ref. 19 to 39): For the 40 to 59 group, the only significant association is a negative one in the Environmental domain (beta = -0.198, p < 0.001). For the 60 to 69 group, a significant negative association exists in the Psychological domain (beta = -0.113, p = 0.023).

* Sex (ref. male): Females show significant positive associations in Physical health (beta = 0.101, p = 0.013) and Psychological domains (beta = 0.100, p = 0.013).

* Education, y (ref. less than or equal to 12): More than 12 years of education is significantly positively associated with all domains (p < 0.001), with the highest beta in Physical health (0.442).

* Monthly household income, won (ref. less than 2,000,000): Higher income brackets (2,000,000 to 4,990,000 and greater than or equal to 5,000,000) generally show significant positive associations across most domains, particularly in the highest bracket where all p-values are less than or equal to 0.001.

Navigate back to Image 2:.

